# Lactosylated *N*-Alkyl polyethylenimine coated iron oxide nanoparticles induced autophagy in mouse dendritic cells

**DOI:** 10.1093/rb/rbx032

**Published:** 2018-01-10

**Authors:** Taipeng Shen, Wencheng Zhu, Li Yang, Li Liu, Rongrong Jin, Jimei Duan, James M Anderson, Hua Ai

**Affiliations:** 1 National Engineering Research Center for Biomaterials, Sichuan University, Chengdu 610065, People’s Republic of China; 2 Shanghai Institute of Biochemistry and Cell Biology, Chinese Academy of Sciences, Shanghai 200031, People’s Republic of China; 3 Departments of Pathology, Macromolecular Science and Biomedical Engineering, Case Western Reserve University, Cleveland, OH 44106, USA; 4 Department of Radiology, West China Hospital, Sichuan University, Chengdu 610065, People’s Republic of China

**Keywords:** dendritic cell, nanoparticle, autophagy, cell maturation

## Abstract

Dendritic cell (DC)-based vaccines have shown promising therapeutic results in cancer and some immune disorders. It is critical to track *in vivo* migration behaviours of DCs and monitor the whole process dynamically and non-invasively. Superparamagnetic iron oxide (SPIO) nanoparticles are chosen for DC labelling under magnetic resonance imaging (MRI) because of their proven biosafety as contrast agents. However, when used for cell labelling, sensitive biological indicators such as cell autophagy may be helpful to better understand the process and improve the probe design. Here, lactosylated *N*-Alkyl polyethylenimine coated SPIO nanoparticles are used for DC labelling. This probe shows satisfactory cell labelling efficiency and low cytotoxicity. In this study, autophagy was used as a key factor to understand how DCs react to nanoparticles after labelling. Our results demonstrate that the nanoparticles can induce protective autophagy in DCs, as inhibition of the autophagy flux could lead to cell death. Meanwhile, the nanoparticles induced autophagy could promote DC maturation which is an essential process for its migration and antigen presentation. Autophagy induced DC maturation is known to enhance the vaccine functions of DCs, therefore, our results suggest that beyond the MRI tracking ability, this probe might enhance therapeutic immune activation as well.

## Introduction

Dendritic cells (DCs) have been recognized as the most potent antigen-presenting cells, thereby triggering T-cell-mediated immune responses [1, 2]. DC-based vaccination for harnessing the potential of a patient’s own immune system has been in trials to treat a variety of diseases [3], including cancers [4], type 1 diabetes mellitus [5] and human immunodeficiency virus-1 infection [6]. Sufficient DCs are required to migrate to lymph nodes for efficient boosting of immune response, but the *in vivo* migration of DCs is quite difficult to monitor. Imaging probes, especially superparamagnetic iron oxide (SPIO) nanoparticles, have been used for labelling of DCs and monitoring their migration to lymphoid tissues under magnetic resonance imaging (MRI) [7]. However, the efficient labelling requires high concentrations of SPIO, up to 200 μg/ml SPIO have been used on immature DCs for tracking [7].

The physical properties of the surface coating materials of SPIOs are key factors determining cellular uptake efficiency and subsequent MR imaging capability [8]. Recent studies show that SPIOs with modifications or combinations significantly expand their applications in multifunctional theranostics [9–15]. Polyethylenimine (PEI) is a widely accepted polycation for gene transfection, as its positive charge is helpful for complexation of negatively charged genes and interactions with the cell membrane. Our previous work shows that the modified PEI/SPIO nanocomposites displayed good performance in cell labelling [16, 17] and gene delivery [18, 19]. More importantly, the modified PEI/SPIO nanocomposites have also exhibited high efficiency and low cytotoxicity on labelling and *in vivo* tracking DCs [20].

Recently, numerous studies have shown that nanoparticles can induce autophagy in different types of cells, including cancer cells [21], lung cells [22], mouse embryonic fibroblasts [23] and human monocytes [24]. Autophagy is a fundamental cellular process, which is responsible for digesting damaged cellular components and foreign materials. Autophagy is activated to ensure cell survival under varied stresses, while massive autophagy leads to cell death. PEI polymer itself has been found to induce autophagy in different cell lines [25, 26], and our recent study shows that lactosylation of PEI can remarkably decrease PEI-induced autophagy and cytotoxicity in RAW 264.7 cells without compromising the MRI capability [27]. However, whether such modified PEI/SPIO nanocomposites are suitable for labelling DCs and how DCs would react to the nanocomposites is unclear.

We have developed lactosylated *N*-Alkyl-PEI coated SPIO nanoparticles with a higher degree of lactose (17.2%) than our previously reported ones (6.8% and 11.7%) [27]. First, we evaluated the MR imaging ability of the nanoparticle, then we assessed the cellular uptake efficiency and cytotoxicity of this nanoparticle towards DCs. Moreover, we investigated how the nanoparticle would influence autophagy, apoptosis and cell maturation in mouse DCs.

## Materials and methods

### Materials

Branched PEI_2k_, 1-iodododecane, ferrozine, neocuproine, ascorbic acid, lactobionic acid (LAC) and dimethylsulfoxide (DMSO) were purchased from Sigma (USA). 1-(3-dimethylaminopropyl)-3-ethylcarbodiimide hydrochloride (EDC) and ammonium acetate were purchased from Aladdin (China). All chemicals were used as received. Balb/c mice were purchased from Chengdu Dashuo Biotechnology Co. Ltd. (China). RPMI-1640, phosphate buffered saline (PBS) and penicillin/streptomycin were purchased from Hyclone (USA). Fetal bovine serum was purchased from Gibco (USA). Granulocyte-macrophage colony-stimulating factor (GM-CSF) was purchased from PeproTech (USA). Lipopolysaccharide (LPS) was purchased from Hycult Biotech (Netherlands). Annexin V-FITC/PI kit and cell counting kit-8 (CCK-8) were purchased from Dojindo (Japan). 3-Methyladenine (3-MA), wortmannin and Mammalian Cell Lysis Reagent were purchased from Sigma (USA). Protease inhibitor cocktail was purchased from Roche (USA). Mouse monoclonal LC3 antibody (NB100-2220) was obtained from Novus (USA), and mouse monoclonal p62 antibody (ab56416) was purchased from Abcam (USA). Mouse monoclonal β-Actin antibody (sc-47778) and goat-anti-mouse-IgG-HRP were purchased from Santa Cruz (USA). Polyvinylidene fluoride (PVDF) membrane and enhanced chemiluminescence (ECL) kit were purchased from Bio-Rad (USA).

### Preparation and characterization of *N*-Alkyl-PEI_2k_-LAC/SPIO

Alkylated branched PEI (*N*-Alkyl-PEI_2k_, 2 kDa) was synthesized following a published method [28]. Briefly, branched PEI_2k_ was reacted with 1-iodododecane in ethanol, then the crude product was dissolved in water, treated with NaOH and dialyzed against water for 2 days. Then *N*-Alkyl-PEI_2k_ was obtained as a gummy solid on freeze-drying. *N*-Alkyl-PEI_2k_ (99 mg, 1.55 mmol) was dissolved in water and LAC (222 mg, 0.62 mmol) was added. Diluted hydrochloric acid and 1-(3-dimethylaminopropyl)-3-EDC (119 mg, 0.62 mmol) were then added dropwise. The mixture was stirred for 3 days at room temperature and the solvent was removed to yield *N*-Alkyl-PEI_2k_-LAC. The product was characterized with ^1 ^H NMR (DMSO) and the grafted ratio was calculated from elemental analysis.

SPIO nanoparticles were synthesized through a high-temperature solution phase reaction following a method from Sun *et al.* [29]. Obtained monodisperse magnetite (Fe_3_O_4_) was stored in n-hexane. SPIO nanoparticles were redispersed in chloroform, after drying under argon gas. Then *N*-Alkyl-PEI_2k_-LAC was dissolved in DMSO, and added into chloroform under sonication, which was mixed with SPIO at a mass ratio of 1 : 0.6. This mixture was added dropwise into water under sonication and kept standing for another 1 h. Finally, the product was purified to get rid of the remaining chloroform and DMSO *via* rotary evaporation and dialysis. Water-soluble *N*-Alkyl-PEI_2k_-LAC/SPIO nanoparticles were characterized before used. Iron concentration was determined by furnace atomic absorption spectroscopy. *T_2_* relaxivity of nanocomposites was determined by a clinical MRI scanner (3.0 T, Siemens).

### Culture of mouse DCs

All animal experiments were performed in compliance with protocols approved by the Institute’s Animal Care and Use Committee. The bone marrow precursors of Balb/c mouse were used to generate DCs as reported [30, 31]. Briefly, at day 0, bone marrow precursors were seeded in a flask with 20 ml RPMI-1640 medium with 20 ng/ml GM-CSF. Then another 20 ml RPMI-1640 medium with 20 ng/ml GM-CSF was added into each flask at day 3. At day 6, a change of half volume of medium was followed. At day 8, loosely adherent cells were harvested. With this protocol, around 2 ∼ 3 × 10^7^ DCs were rendered per mouse. For autophagy analysis, the immature DCs at a concentration of 1 × 10^6^ cells/ml were incubated with *N*-Alkyl-PEI_2k_-LAC/SPIO over a certain period of time. In some experiments, cells were first incubated with medium containing autophagy inhibitors for 2 h in a CO_2_ incubator and then were exposed to fresh medium containing desired amounts of *N*-Alkyl-PEI_2k_-LAC/SPIO.

### Cellular uptake of *N*-Alkyl-PEI_2k_-LAC/SPIO

Intracellular iron content was determined using the ferrozine assay [32]. After 12 h incubation with desired doses of *N*-Alkyl-PEI_2k_-LAC/SPIO nanoparticles, DCs, seeded in 6-well plates at 1 × 10^6^ cell/ml, were collected and washed with PBS twice. Cells were resuspended in 100 μl of 0.05 mM NaOH for 2 h. Then the samples were mixed with 100 μl 0.01 mM HCl and 100 μl of freshly prepared oxidant (4.5% KMnO_4_ and 1.4 mM HCl mixed at equal volumes) and incubated at 60 °C for 2 h with protection from light. The reaction mixtures were cooled down to RT before the addition of the iron reaction reagent (6.5 mM ferrozine, 13.1 mM neocuproine, 1 M ascorbic acid, 2.5 M ammonium acetate dissolved in water). They were then incubated for 30 min on a shaker, and measured with a microplate reader at 570 nm (Bio-Rad, USA). The concentrations were calculated according to a standard curve as described before [20, 33].

### CCK-8 assay

Cell viability of DCs was measured by a standard CCK-8 assay following the manufacturer’s instructions. DCs were seeded in 96-well plates (1 × 10^4^ cells per well, 100 µl) and incubated in culture medium with *N*-Alkyl-PEI_2k_-LAC/SPIO at different Fe concentrations for 12 h. 10 µl CCK-8 solutions were added to each well of the plates and the cells were incubated for 2 h in a CO_2_ incubator. Absorbance at 450 nm was measured through a microplate reader (Bio-Rad, USA).

### Western blot analysis

DCs were seeded in 12-well plates (1 × 10^6^ cells per well) with *N*-Alkyl-PEI_2k_-LAC/SPIO (Fe: 5, 10 μg/ml) for 12 h in a CO_2_ incubator. The cells were harvested and lysed in Mammalian Cell Lysis Reagent with protease inhibitor cocktail. An equal amount of protein (20 μg) for each sample was subjected to SDS-PAGE (15% or 13.3% separation gels) and transferred to a PVDF membrane (Bio-Rad, USA). After blocking with 5% non-fat milk in PBST (PBS containing 0.5% Triton-X 100) at room temperature for 1 h, membranes were washed three times with PBST and incubated overnight at 4 °C with primary antibodies with constant gentle shaking. The membranes were washed three times with PBST, followed by 1 h incubation at room temperature with secondary antibodies. Membranes were washed three times in PBST. The antigen-antibody complexes were visualized with an ECL kit.

### Transmission electron microscopy assay

DCs at a concentration of 2 × 10^5^ cells/ml were incubated with or without *N*-Alkyl-PEI_2k_-LAC/SPIO (Fe: 10 μg/ml) for 12 h. Cells were harvested and prepared for TEM analysis as described before [20, 33]. Simply, DCs were collected, washed with PBS and immediately fixed in 4% glutaraldehyde for at least 1 day. The samples then were washed three times with 0.1 M PBS and post-fixed with 1% osmic acid for 2 h at room temperature. Finally, they were dehydrated serially with 50%, 70%, 80%, 90% and 100% alcohol and 100% acetone, and embedded in epoxy resin overnight for microtome sectioning. Ultrathin sections were stained with 2% uranyl acetate and lead citrate for 15 min, respectively, and analysed with TEM (Hitachi HT7700, Japan).

### Flow cytometry analyses for apoptosis and surface markers

Cell apoptosis was detected with an annexin V-FITC/PI kit following the manufacturer’s instruction. DCs were seeded in 6-well plates (1 × 10^6^ cells per well) and treated with *N*-Alkyl-PEI_2k_-LAC/SPIO (Fe: 10 μg/ml) or wortmannin for 12 h in a CO_2_ incubator. The cells of interest were collected by centrifugation, washed with PBS, and resuspended at 10^6^ cell/ml with 1 × annexin V binding buffer. Then 5 μl annexin V-FITC conjugates and 5 μl propidium iodide (PI) solution were added and incubated for 15 min in the dark. Finally, the cell suspension was diluted to a final volume of 500 μl/assay tube with 1 × annexin V binding buffer and analysed within 1 h by flow cytometric analysis (BD FACS Aria SORP, USA). At least 30 000 cells were analysed to determine the percentage of apoptotic cells.

DCs were treated with *N*-Alkyl-PEI_2k_-LAC/SPIO (10 μg/ml) with or without 3-MA (2 mM) or LPS (1 μg/ml) for 12 h. For surface markers staining, 10^6^ cells per sample were incubated in 100 μl PBS containing 2 μl monoclonal antibodies for 30 min at 4 °C, washed twice with PBS and analysed by fluorescence activated cell sorting (FACS) (BD FACSAria SORP, USA). Phycoerythrin (PE)-conjugated anti-CD11c (HL3) and PE-conjugated anti-CD80 (16-10A1) antibodies were used for detecting DC surface markers. At least 30 000 cells were analysed to determine the percentage of positive cells.

### Statistical analysis

At least three samples were used for data analysis in each set of experiments. The data presented as mean ± standard deviations (SD). One-way analysis of variance was used for group means testing while a student’s *t*-test was used to compare the means of two samples. *P *<* *0.05 was considered as significant.

## Results and discussion

### Characterization of *N*-Alkyl-PEI_2k_-LAC/SPIO nanoparticles

**Figure 1:**
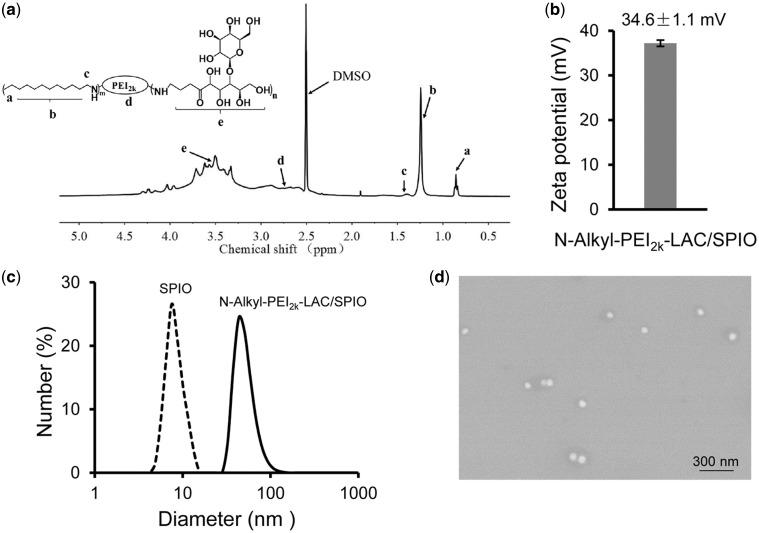
(**a**) ^1 ^H NMR spectrum of *N*-Alkyl-PEI_2k_-LAC (DMSO), characteristic peaks are identified by arrows. (**b**) Zeta potential of *N*-Alkyl-PEI_2k_-LAC/SPIO nanoparticles. (**c**) DLS of SPIO and *N*-Alkyl-PEI_2k_-LAC/SPIO nanoparticles. SPIO showed a diameter of 8.7 ± 0.6 nm in n-hexane, *N*-Alkyl-PEI_2k_-LAC/SPIO showed a diameter of 57.4 ± 4.5 nm. (**d**) SEM of *N*-Alkyl-PEI_2k_-LAC/SPIO nanoparticles

For clinical applications, conventional SPIO nanoparticles must be modified with polymers to obtain colloidal stability, function and biocompatibility. Amphipathic PEI was synthesized by grafting with iododecane and modified with LAC to improve its biocompatibility. Chemical structures of amphiphilic *N*-Alkyl-PEI_2K_ and *N*-Alkyl-PEI_2k_-LAC were confirmed by ^1 ^H NMR (Fig. 1a) and elemental analysis. And calculated upon elemental analysis results, the grafting ratio of Alkyl was 9.3%, while the grafting ratio of LAC was 17.2%. *N*-Alkyl-PEI_2k_-LAC (400 MHz, DMSO): δ 4.74–3.12 (LAC), 3.11–2.30 (–NH–CH_2_–CH_2_–NH–), 1.34–1.07 (–CH_2_–(CH_2_)_10_CH_3_), 0.85 (–CH_2_–(CH_2_)_10_CH_3_). Our recent study shows that lactosylation of PEI can reduce PEI-caused cytotoxicity with an increase of lactosylation degrees and, in this report, the lactosylation degree is higher than (6.8% and 11.7%) reported previously [27], suggesting a possible lower cytotoxicity of the newly developed nanocomposites. Among these nanoparticles, the grafting degrees of LAC were controlled by regulating the feed ratio between LAC and PEI in reaction.

The monodispersed SPIO nanocrystals were dispersed in n-hexane and amphiphilic *N*-Alkyl-PEI_2k_-LAC can transfer the hydrophobic SPIO nanocrystals into a water phase. Size has a great impact on cellular uptake of polymer complexes. Figure 1c shows that the SPIO has a relatively narrow size distribution in n-hexane, with a mean diameter of 8.7 ± 0.6 nm characterized by DLS. *N*-Alkyl-PEI_2k_-LAC/SPIO shows a diameter of 57.4 ± 4.5 nm in water. The surface charge of *N*-Alkyl-PEI_2k_-LAC/SPIO is positive (zeta potential = +34.6 ± 1.1 mV) (Fig. 1b) which is lower than the charge of *N*-Alkyl-PEI_2k_ PEI coated nanoparticles (zeta potential around +40 mV) [16]. Scanning electron microscopy (SEM) shows that the morphology of the dry sample features spherical particles with homogeneous dimension (Fig. 1d), indicating that this nanocomposite was well dispersed in water without obvious aggregation.

### *T_2_* relaxivity of *N*-Alkyl-PEI_2k_-LAC/SPIO nanoparticles

**Figure 2:**
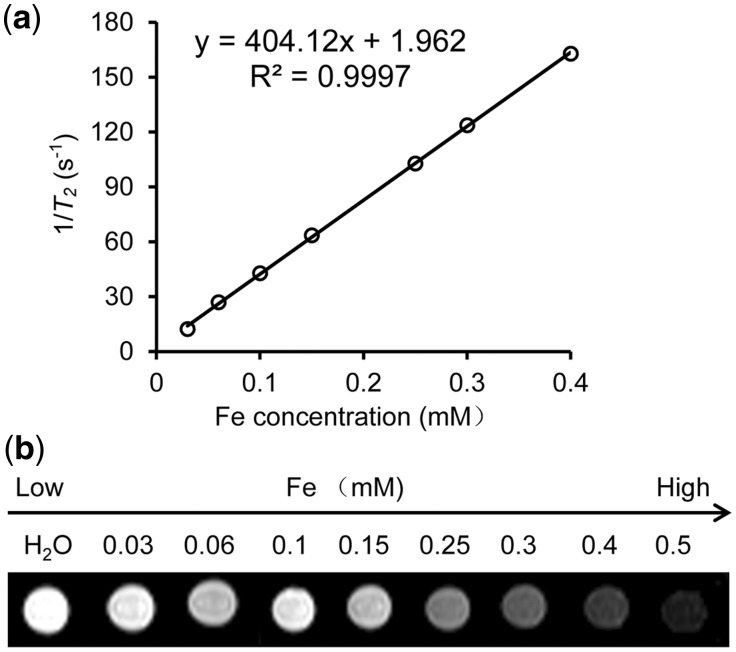
(**a**) *T_2_* Relaxation rate (1/*T_2_*, s^−1^) as a function of Fe concentration (mM) for *N*-Alkyl-PEI_2k_-LAC/SPIO nanoparticles at 3.0 T. (**b**) *T_2_*-weighted MRI images of *N*-Alkyl-PEI_2k_-LAC/SPIO nanoparticles at varied concentrations in water (3.0 T, spin-echo sequence: TR = 5000 ms, TE = 20 ms)

Previous works have demonstrated that nanocomposites containing multiple SPIO nanocrystals show higher *T_2_* relaxivities than those containing single SPIO nanocrystals [28]. A probable explanation is that nanoparticles hold an increased magnetic moment in an aqueous solution. As a result, a high *T_2_* relaxivity of the *N*-Alkyl-PEI_2k_-LAC/SPIO nanoparticles (404.12 Fe mM^−1 ^s^−1^) was detected at 3.0 T magnetic field (Fig. 2). This imaging capability indicates that this probe could act as a good MR imaging contrast agent.

### Intracellular Fe content

**Figure 3:**
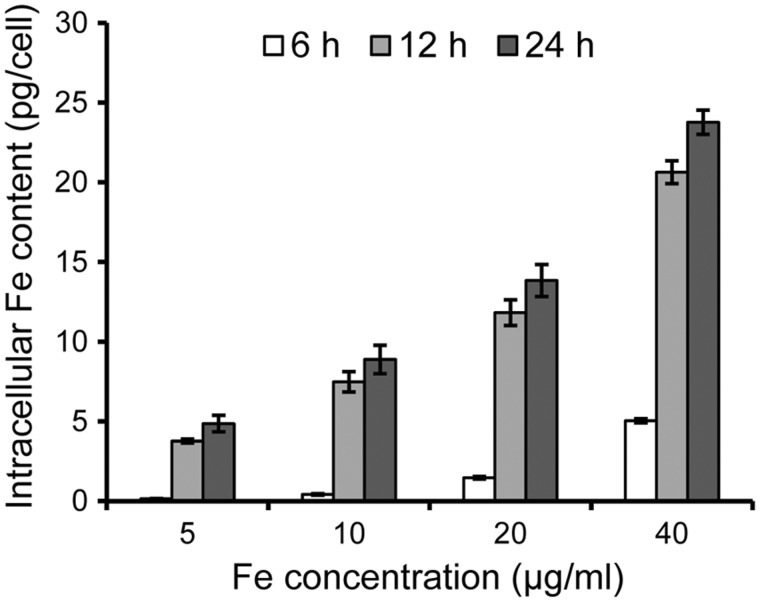
Intracellular iron content of *N*-Alkyl-PEI_2k_-LAC/SPIO nanoparticles labelled DCs. The iron content of DCs after labelling with *N*-Alkyl-PEI_2k_-LAC/SPIO nanoparticles for 6, 12 or 24 h with varied iron concentrations

**Figure 4:**
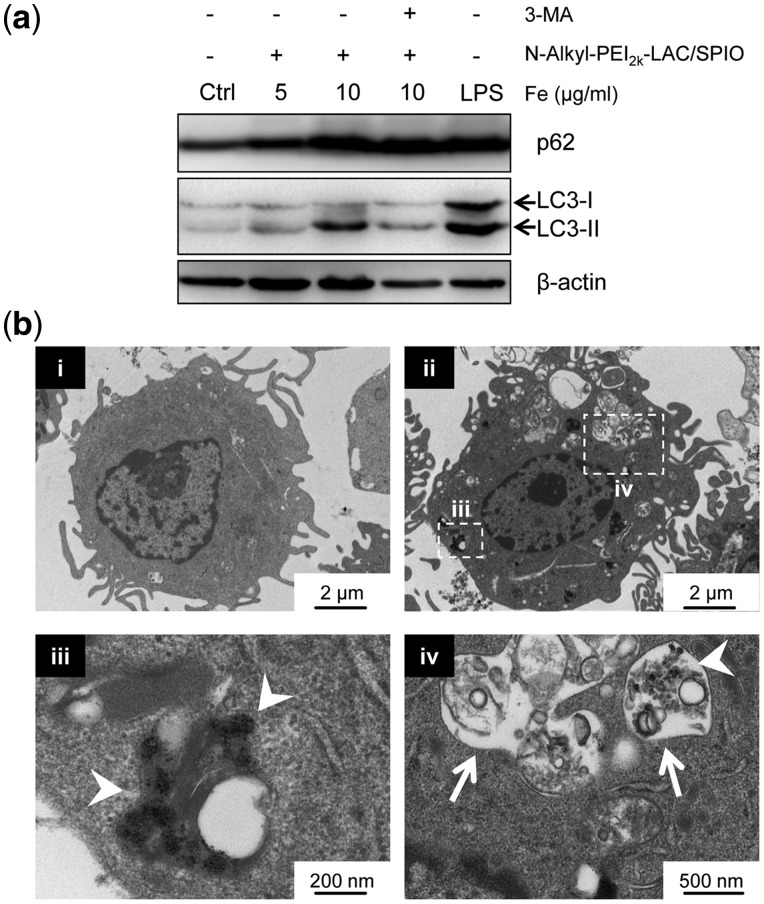
*N*-Alkyl-PEI_2k_-LAC/SPIO nanoparticles induce protective autophagy in DCs. (**a**) Western blotting assay of DCs that were untreated (negative control), treated with nanoparticles (5, 10 μg/ml, 12 h) or LPS (1 μg/ml, 12 h, positive control). 3-MA (2 mM, pre-treatment with DCs for 2 h) is a classic autophagy inhibitor. (**b**) TEM of untreated DCs (control) and nanoparticle treated DCs (10 μg/ml, 12 h). The lower figures (iii and iv) are the enlarged regions from the upper figure (ii). Intracellular accumulation of SPIO nanoparticles in vesicles (white arrowheads), and autophagic vesicle formation (white arrows)

To test its capability as a cell labelling probe, we used *N*-Alkyl-PEI_2k_-LAC/SPIO nanoparticles to label mouse DCs. The intracellular iron content was measured after DCs were labelled with the nanoparticles under different conditions. As shown in Fig. 3, the uptake of nanoparticles by DCs presents a time- and dose-dependent mode, similar to our previous reports [20, 33]. Higher Fe concentration, longer time or both would generate a higher level of intracellular nanoparticle uptake by DCs. After a 12 h incubation with 10 μg/ml Fe in cell suspension, the internalized iron content per cell was about 8 μg/cell which is close to our recent report under the same labelling condition [20]. This labelling condition was thus chosen for the following experiments. The TEM images (Fig. 4b, ii) show that the nanoparticles were dispersed throughout the cytoplasm as dense agglomerate bodies, indicating they were phagocytosed by DCs, whereas the untreated DCs did not have such fuscous granules (Fig. 4b, i). Together, these results demonstrate that *N*-Alkyl-PEI_2k_-LAC/SPIO nanoparticles possess a high labelling efficiency towards DCs.

### *N*-Alkyl-PEI_2k_-LAC/SPIO nanoparticles induce autophagy in DCs

Previous reports have demonstrated that PEI itself could induce autophagy in nephritic and hepatic cell lines [25]. Our recent study indicates that lactose-modified PEI coated SPIO nanoparticles could significantly reduce PEI-caused autophagy and cytotoxicity in RAW 264.7 cells [27]. To determine how DCs would respond to *N*-Alkyl-PEI_2k_-LAC/SPIO nanoparticles, we detected the autophagy flux after treatment with nanoparticles. First, we investigated LC3 conversion (from LC3-I to LC3-II) by immunoblotting, as it is a well-established marker of autophagy [34]. Compared to untreated DCs, LC3-II with iron concentrations increased blocked by pre-treatment with 3-methyladenine (3-MA), a classic autophagy inhibitor [35], as shown in Fig. 4a. Meanwhile, we detected the protein level of p62, because its degradation is associated with the progress of autophagy [36]. However, the protein level of p62 showed an increase instead of decrease, which indicates that the positive charges of PEI might deactivate the lysosomes by elevating lysosomal pH. Also, it is possible that the nanoparticles may activate the formation of an autophagosome-like structure through an increase in p62 [37].

In addition, transmission electron microscopy (TEM) was applied to observe the formation of autophagic vesicles in DCs. One of the key criteria for autolysosomes is the damaged organelles and other undigested materials contained in the double membrane structures [38]. After treatment with *N*-Alkyl-PEI_2k_-LAC/SPIO (10 µg/ml) for 12 h, we observed an obvious increase of autophagosomes in DCs (Fig. 4b, iv, white arrows) compared with the control group (Fig. 4b, i). The black dots in DC’s cytoplasm indicated high electronic density SPIO nanoparticles (Fig. 4b, iii and iv, white arrow heads). Therefore, we concluded that *N*-Alkyl-PEI_2k_-LAC/SPIO nanoparticles can induce autophagy in DCs.

### *N*-Alkyl-PEI_2k_-LAC/SPIO nanoparticles induce protective autophagy in DCs

**Figure 5:**
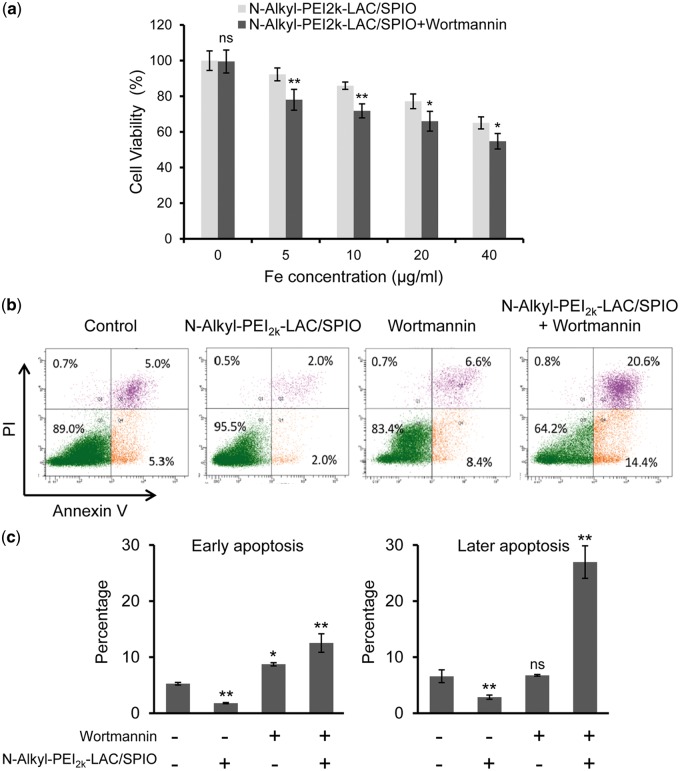
Roles of *N*-Alkyl-PEI_2k_-LAC/SPIO nanoparticle-induced autophagy in DCs. (**a**) Cell viability of *N*-Alkyl-PEI_2k_-LAC/SPIO nanoparticle labelled DCs was evaluated with a CCK-8 assay. For wortmannin treatment, DCs were pre-treated with the drug (50 nM) for 2 h. Results were represented as the mean ± SD, *n* = 5; *P <* *0.05, **P < 0.01. (**b**) Cells were pre-treated with or without wortmannin (50 nM) and incubated with the nanoparticles (10 μg/ml) for 12 h. Cell apoptosis was detected using an annexin V-FITC/PI kit. Viable cells (annexin V- and PI-), early apoptotic cells (annexin V+ and PI-), late apoptotic cells and necrotic cells (annexin V+ and PI+) and damaged cells (annexin V- and PI+) are located in the bottom left, bottom right, top right and top left quadrants, respectively. Numbers indicate percent of cells in each quadrant. (**c**) Percentages of apoptotic cells in each group. The results were obtained from three independent experiments and at least 30 000 cells were analysed in each experiment. *P <* *0.05, **P <* *0.01

As *N*-Alkyl-PEI_2k_-LAC/SPIO nanoparticles could induce autophagy in DCs, we tried to understand the biological significance of the nanoparticle-induced autophagy in DCs. We used a standard CCK-8 assay to measure the cytotoxicity of nanoparticles for DCs. As shown in Fig. 5a, cell viability decreased with increasing iron concentrations indicating this nanoparticle has a certain degree of cytotoxicity. Even so, when the iron concentration was 10 µg/ml or lower, the nanoparticle showed no obvious cytotoxicity. Moreover, according to our recent study, this labelling condition could show a satisfactory *in vivo* MRI outcome [20]. However, inhibition of autophagy by wortmannin, a non-specific covalent inhibitor of phosphatidylinositol 3-kinase influencing the formation of autophagosome [39], could lead to cell death (Fig. 5a). These data suggest that the *N*-Alkyl-PEI_2k_-LAC/SPIO nanoparticle- induced autophagy plays a positive role in DCs.

PEI itself is known to induce cell death through apoptosis [40]. Apoptosis refers to a cell intrinsic mechanism for suicide which is controlled by a variety of cellular signalling pathways. We tested the apoptosis process in DCs after treatment with *N*-Alkyl-PEI_2k_-LAC/SPIO nanoparticles with an annexin V/PI assay. At the early stages of apoptosis, phosphatidylserine residues are externalized to the outer plasma membrane, which can be combined with annexin V-FITC conjugates to be detected, otherwise PI is used as a DNA stain to differentiate necrotic, apoptotic and healthy cells. The data in Fig. 5a and c show that inhibiting *N*-Alkyl-PEI_2k_-LAC/SPIO nanoparticle-induced autophagy by wortmannin could significantly increase the ratio of both early and later apoptotic cells. Wortmannin alone could slightly increase the number of early apoptotic cells. Interestingly, *N*-Alkyl-PEI_2k_-LAC/SPIO nanoparticles could reduce the apoptotic cells in DCs compared to untreated DCs, suggesting that induced autophagy might prolong the lifetime of DCs as the matured DCs are not proliferative. These results demonstrate that the autophagy induced by *N*-Alkyl-PEI_2k_-LAC/SPIO in DCs plays a positive role in reducing nanoparticle-induced cytotoxicity and thus prevents apoptotic death, in accordance with the CCK-8 assay results.

### *N*-Alkyl-PEI_2k_-LAC/SPIO nanoparticles induced autophagy promotes DC maturation

**Figure 6:**
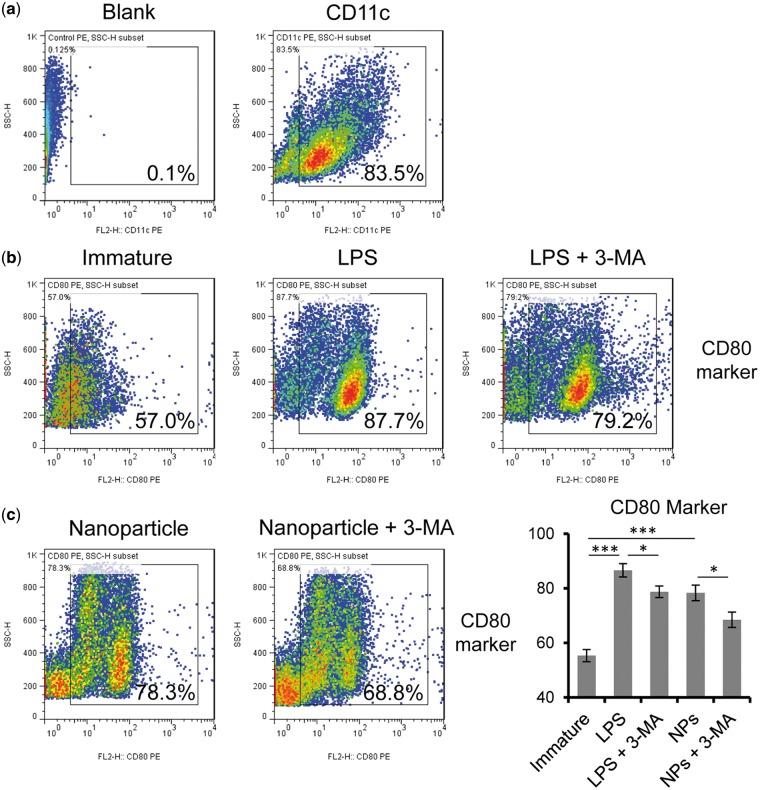
*N*-Alkyl-PEI_2k_-LAC/SPIO nanoparticle-induced autophagy promote DCs maturation. (**a**) CD11c marker was used to verify the purity of cultured DCs. (**b**) LPS (1 μg/ml, 12 h) was used to induce autophagy dependent cell maturation which serves as a positive control. (**c**) DCs were incubated with *N*-Alkyl-PEI_2k_-LAC/SPIO nanoparticles (10 μg/ml) for 12 h, pre-treated with 3-MA for 2 h followed by the indicated stimulations. CD80 was analysed as a cell maturation surface marker in DCs. (**d**) Percentages of CD80+ DCs in each group. NPs indicate the *N*-Alkyl-PEI_2k_-LAC/SPIO nanoparticles. The results are obtained from three independent experiments and at least 30 000 cells were analysed in each experiment. *P <* *0.05, ***P <* *0.001

**Figure 7:**
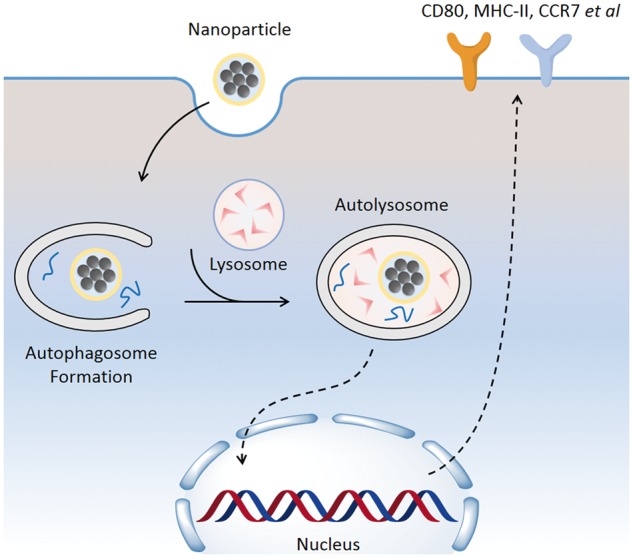
A schematic model showing that *N*-Alkyl-PEI_2k_-LAC/SPIO nanoparticles induced autophagy and maturation in mouse DCs. First, the internalized *N*-Alkyl-PEI_2k_-LAC/SPIO nanoparticles could activate autophagy which plays a protective role in cell survival. Then, the autophagy flux triggers cellular signalling, thereby activating the expression of DC maturation related genes. Eventually, the surface makers of matured DCs show a dramatic increase

Autophagy is an essential signalling pathway in many cellular events. As for DCs, autophagy is required for processing the engulfed antigens and promoting cell maturation [41, 42]. Hence, we analysed DC maturation by detecting the expression of surface markers through FACS. CD11c is a representative marker for DCs [43], which indicates the purity of harvested cells. As shown in Fig. 6a, over 83% are CD11c + DCs. Several markers are used to monitor DC maturation, including CD80, CD 86, MHC-II and CCR7 [43, 44]. Here, we used CD80 as a maturation marker and found that LPS could strongly increase DC CD80+ levels to 87.7%, while in the untreated group the CD80+ DCs is 57.0% (Fig. 6b). This result is consistent with previous studies that LPS induced autophagy promotes DC maturation [42, 45]. The autophagy inhibitor 3-MA could partially reduce the percentage to 79.2% (Fig. 6b). We detected an increase of CD80+ DCs in *N*-Alkyl-PEI_2k_-LAC/SPIO nanoparticle treated DCs which is 78.3%. More importantly, with the pre-treatment of 3-MA, the nanoparticle induced CD80+ DCs decreased to 68.8%. Figure 6d summarizes how the CD80+ DC populations changed after different treatments. Our recent report on glycidol modified PEI/SPIO nanoparticles shows that the nanoparticles combined with LPS and TNF-α could further enhance DC maturation, compared to the induced maturation by LPS and TNF-α alone [20]. It is noteworthy that the nanoparticle-induced autophagy in DCs might strengthen their vaccine function, according to a previous publication [46]. As we summarized in Fig. 7, the results indicate that endocytosed *N*-Alkyl-PEI_2k_-LAC/SPIO nanoparticle triggers autophagy which further contributes to maturation in DCs.

## Conclusions

In this study, we used *N*-Alkyl-PEI_2k_-LAC/SPIO nanoparticles to label DCs as a MR contract agent, and investigated how the nanoparticles would affect DCs. This nanoparticle displays a high *T_2_* relaxivity (404.12 Fe mM^−1 ^s^−1^) under a clinical 3T scanner. It can label DCs with high efficiency, as at the concentration of 10 mg Fe/ml, the intracellular iron content is sufficient for MR imaging. This nanoparticle shows low cytotoxicity towards DCs, indicating it is a promising MR probe for cell labelling. In addition, we found that the nanoparticle can induce autophagy and inhibition of the autophagy could lead to apoptotic cell death, suggesting this nanoparticle-induced autophagy is protective in DCs. Furthermore, we found that *N*-Alkyl-PEI_2k_-LAC/SPIO nanoparticle-caused autophagy contributes to DC maturation. As the enhanced DC maturation could elevate the antigen presenting abilities, therefore, beyond its MR imaging capability, this nanoparticle might also participate in boosting the immune activation in DC-based vaccines.

*Conflict of interest statement*. None declared.

## Funding

This work was supported by grants from National Key Basic Research Program of China (2013CB933903), and National Natural Science Foundation of China (81621003, 20974065, 51173117 and 50830107).
